# Inhibition of TNF Receptor p55 By a Domain Antibody Attenuates the Initial Phase of Acid-Induced Lung Injury in Mice

**DOI:** 10.3389/fimmu.2017.00128

**Published:** 2017-02-13

**Authors:** Michael R. Wilson, Kenji Wakabayashi, Szabolcs Bertok, Charlotte M. Oakley, Brijesh V. Patel, Kieran P. O’Dea, Joanna C. Cordy, Peter J. Morley, Andrew I. Bayliffe, Masao Takata

**Affiliations:** ^1^Section of Anaesthetics, Pain Medicine and Intensive Care, Faculty of Medicine, Imperial College London, Chelsea and Westminster Hospital, London, UK; ^2^Department of Intensive Care Medicine, Tokyo Medical and Dental University, Tokyo, Japan; ^3^Biopharm Molecular Discovery, GlaxoSmithKline R&D, Stevenage, UK

**Keywords:** CD120a, TNFRSF1a, acid aspiration, inflammation, respiratory mechanics

## Abstract

**Background:**

Tumor necrosis factor-α (TNF) is strongly implicated in the development of acute respiratory distress syndrome (ARDS), but its potential as a therapeutic target has been hampered by its complex biology. TNF signals through two receptors, p55 and p75, which play differential roles in pulmonary edema formation during ARDS. We have recently shown that inhibition of p55 by a novel domain antibody (dAb™) attenuated ventilator-induced lung injury. In the current study, we explored the efficacy of this antibody in mouse models of acid-induced lung injury to investigate the longer consequences of treatment.

**Methods:**

We employed two acid-induced injury models, an acute ventilated model and a resolving spontaneously breathing model. C57BL/6 mice were pretreated intratracheally or intranasally with p55-targeting dAb or non-targeting “dummy” dAb, 1 or 4 h before acid instillation.

**Results:**

Acid instillation in the dummy dAb group caused hypoxemia, increased respiratory system elastance, pulmonary inflammation, and edema in both the ventilated and resolving models. Pretreatment with p55-targeting dAb significantly attenuated physiological markers of ARDS in both models. p55-targeting dAb also attenuated pulmonary inflammation in the ventilated model, with signs that altered cytokine production and leukocyte recruitment persisted beyond the very acute phase.

**Conclusion:**

These results demonstrate that the p55-targeting dAb attenuates lung injury and edema formation in models of ARDS induced by acid aspiration, with protection from a single dose lasting up to 24 h. Together with our previous data, the current study lends support toward the clinical targeting of p55 for patients with, or at risk of ARDS.

## Introduction

Acute respiratory distress syndrome (ARDS) is a major cause of patient morbidity and mortality within the ICU, constituting ~10% of ICU admissions worldwide with an associated mortality of 30–50% (1). ARDS can result from various insults, of which aspiration of acidic gastric contents is a major contributor both within the community and in the operating theater during anesthesia (2). Inflammation has been considered to be key in developing ARDS, but attempts to minimize “global” inflammation, e.g., by use of corticosteroids have delivered limited mortality benefits (3–7), suggesting the need for a more targeted approach. Among all potential targets, tumor necrosis factor-α (TNF) is one of the strongest candidates for such interventions—it is one of the earliest expressed “gate-keeper” cytokines in response to almost any potentially damaging situation, modulating subsequent inflammatory responses, and has been repeatedly implicated in the development and progression of ARDS (8), including direct effects on pulmonary edema formation and clearance. Despite this, previous clinical trials of anti-TNF therapy have shown little beneficial impact within the ICU setting (9). This may be partly attributed to the biophysical properties of the inhibitors used (e.g., affinity and tissue penetration), and also likely related to the complex nature of TNF signaling.

Tumor necrosis factor signals through two cell surface receptors, TNF receptor (TNFR) type I (p55) and TNFR type II (p75). Historically, p75 signaling was considered to be adjunct to the p55 pathway (10, 11), but it is now becoming clear that each TNFR subtype has signaling capabilities of its own, which in particular circumstances may lead to directly opposing consequences (12–16). In line with this, we have previously shown using genetically modified mice that specific absence of p55 is protective in the very acute phase of ARDS induced by mechanical ventilation (17), while absence of p75 seems to be detrimental. We have also found that p55 signaling triggers alveolar epithelial cell dysfunction in the early phase of ARDS, promoting lung permeability as well as impairing alveolar fluid reabsorption (18). These findings suggest that while total TNF signaling blockade would likely be counterproductive, specific therapeutic strategies targeting the individual TNFRs could be effective in ARDS.

We have recently demonstrated efficacy of selective pharmacological blockade of p55 signaling in an acute model of mouse ventilator-induced lung injury (VILI), using a novel IgG fragment known as a domain antibody (Biopharmaceuticals R&D, GlaxoSmithKline, Stevenage, UK) (19). Domain antibodies (dAb™) offer multiple advantages over conventional antibody technology (20)—for example, they have fewer off-target effects and can be delivered at much higher concentrations per unit mass compared to conventional antibodies due to the lack of an Fc region and can be manufactured to be suitable for local delivery such as inhalation. In the current study, we tested the efficacy of a dAb antagonist of murine p55, in mouse models of acute and resolving acid aspiration-induced lung injury (21). This enabled us to both evaluate the acute benefits of pharmacological p55 blockade in a highly clinically relevant model and explore whether any beneficial effects persisted beyond the acute stage. A similar dAb antagonist of human p55 has recently entered early clinical development for lung injury (22).

## Materials and Methods

All protocols were approved by the Ethical Review Board of Imperial College London and carried out under the authority of the UK Home Office in accordance with the Animals (Scientific Procedures) Act 1986, UK. Male C57BL/6 mice (Charles River, Margate, UK) aged 9–12 weeks old, weighing 25–30 g were used throughout.

The p55-targeting dAb sequence was identified from a phage display library. To determine binding kinetics, murine p55 or p75 was immobilized on an IgG surface, varying concentrations of dAb (from 0.25 to 16 nM) were passed over the surface, and interactions were evaluated via surface plasmon resonance using a Biacore T200 system. Association constant (ka) of the dAb for p55 was determined as 3.455 × 10^7^ (1/Ms) and dissociation constant (kd) was 0.0014 (1/s), indicating high affinity of binding (KD) of 4.05 × 10^−11^M, assuming a 1:1 binding stoichiometry. In contrast, no specific binding was observed to p75.

### Ventilated Acid Aspiration Model

We have previously shown that genetic absence of TNFR p55 signaling attenuates pulmonary edema formation during the first 2–3 h after acid instillation in mice (18). Therefore, initial experiments were carried out to determine whether p55 dAb administration would have a similar influence during the acute phase of acid aspiration-induced lung injury.

Mice were anesthetized (intraperitoneal ketamine 80 mg/kg and xylazine 8 mg/kg), tracheostomized, and connected to a custom-made ventilator/pulmonary function testing system as described previously (23). Animals had a cannula placed in the carotid artery for monitoring of blood pressure, blood gas analysis, and fluid replacement. All animals were ventilated using 7 ml/kg tidal volume, 2.5 cmH_2_O positive end-expiratory pressure (PEEP), and respiratory rate of 120/min, with 100% O_2_. Following instrumentation, either specific murine TNFR p55 blocking dAb (Dom-1m-15-12, GSK) or non-targeting control dAb (“dummy”) was intratracheally delivered in bolus form (25 µg of antibody in a volume of 50 µl) via a fine cannula passed through the endotracheal tube. The lungs were then immediately recruited with four sustained inflation maneuvers (35 cmH_2_O, 5 s) to maximize distribution of the antibody into the lungs. Mice were ventilated for 1 h to allow respiratory mechanics to return toward normal, and then 65 µl of 0.075M hydrochloric acid (HCl) was instilled into the trachea through the endotracheal tube. Lungs were again recruited by sustained inflations, and mice were ventilated for a further 3 h. Anesthesia was maintained by bolus administrations of intraperitoneal ketamine (40 mg/kg) and xylazine (4 mg/kg) every 20–25 min.

Airway pressure and arterial blood pressure were monitored continuously. Plateau pressure, and respiratory system elastance and resistance were determined every 20 min by the end-inflation occlusion technique, followed each time by sustained inflation (35 cmH_2_O for 5 s) to avoid the development of atelectasis (23). Arterial blood gases were assessed at predetermined points throughout the protocol (immediately before, 60, 120, and 180 min after acid instillation). At the end of the experiments, animals were exsanguinated, lung lavage was performed using 750 µl of saline, and lung tissue samples were taken for further analysis. Each animal was experimented on a separate day, so each observation reflects an independent experiment.

### Spontaneously Breathing Model of Acid Aspiration

While the use of mechanical ventilation provides many advantages, including real-time cardiorespiratory monitoring and an ability to directly compare findings with those of our previous studies in genetically modified animals (18), by definition the model is limited to investigation within the acute phase. We have recently developed a spontaneously resolving model of acid aspiration-induced lung injury (21), which, unlike a number of other models, mimics most of the features of clinical ARDS over up to 5–10 days (24–26). We therefore employed this resolving model to investigate the impact of intranasally delivered p55-targeting dAb on the later phases of injury/start of the repair process.

All animals were anesthetized briefly with inhalational isoflurane (2%) and intranasally dosed with 100 µg (50 µl total volume, divided into two nostrils) of either p55-targeting dAb or dummy dAb. Four hours after dosing (a period designed to allow distribution of the antibody and full recovery of respiratory mechanics from the nasal dosing procedure under non-ventilated conditions), animals were reanesthetized either for physiological analysis (see below—0 h mice) or for acid instillation according to our previously published protocols (21). In brief, mice were suspended vertically for orotracheal instillation, and a fine catheter passed through the vocal cords. A total of 75 µl of an isoosmolar solution of 0.1M hydrochloric acid (pH 1.0) was instilled, and mice received an intraperitoneal bolus of 0.9% saline for fluid resuscitation. Mice were maintained in a humidified chamber containing supplemental oxygen (decreasing from FiO_2_ 1.0–0.4) over the next 4 h and were then returned to individually ventilated cages. Due to the nature of the dosing technique and postdosing care requirements, a maximum of three animals were dosed with acid/antibody on a single day. Each set of data shown in the Section “Results,” of any given combination of treatment (dummy versus p55-targeting dAb) and time point (0, 24, 48, and 72 h), therefore represents observations from 5 to 8 mice, obtained from 3 to 4 independent experiments.

Physiological analysis was carried out at predetermined end points, i.e., 0, 24, 48, or 72 h post acid. Mice were anesthetized (ketamine 80 mg/kg, xylazine 8 mg/kg) and instrumented as described for the acute ventilated model. Immediately after completing surgical preparation, lungs were recruited by sustained inflation (35 cmH_2_O for 5 s). Mice were then ventilated with 7 ml/kg tidal volume, 2.5 cmH_2_O PEEP, and respiratory rate of 120/min using 100% O_2_ for 30 min, in order to standardize the volume history of the lung and determine the PaO_2_/FiO_2_ ratio from carotid blood samples (21). At the end of 30 min ventilation, respiratory mechanics and blood gases were evaluated, and animals were terminated by exsanguination. The right lung was tied off at the hilum, weighed, and placed in an oven at 60°C for determination of wet:dry weight ratio. The left lung was lavaged using 400 µl saline and dissected out for further analysis.

### Evaluation of Injury and Inflammation in Lavage Samples

Total protein concentration in lung lavage fluid was measured as an indicator of alveolar-capillary permeability (Bio-Rad, Hertfordshire, UK). Concentrations of interleukin 6 (IL-6), the neutrophil chemoattractants CXCL1 and CXCL2, and the monocyte chemoattractant CCL2 were measured by ELISA (R&D Systems, Abingdon, UK). The number of neutrophils in lavage fluid was determined by microscopic cytology using hemocytometer and Cytospin-prepared slides.

### Lung Tissue Flow Cytometry

The identification and quantification of leukocytes within lung tissue was performed by flow cytometry using methods described and validated previously. For the acute ventilated model, lung samples were removed, minced, and passed through a 40 µm filter (19, 27). Samples were resuspended in a washing buffer (PBS with 2% FCS, 0.1% sodium azide, and 5 mM EDTA) and stained for 30 min in the dark at 4°C with fluorophore-conjugated anti-mouse antibodies for CD11b (clone M1/70), Gr-1 (RB6-8C5) (both BD BioSciences), and F4/80 (CI:A3-1) (Biolegend). For the spontaneously breathing resolution model, the potential for influx of other cell types over time led us to modify this approach for further identification of leukocyte subpopulations (18). Lung tissues were excised and fixed with Cytofix/Cytoperm (BD Biosciences, Oxford, UK), mechanically disrupted by gentleMACS Dissociator (Miltenyi Biotech, Surrey, UK) and passed through a 40 µm filter to prepare single-cell suspensions. Samples were then stained for 30 min in the dark at 4°C with fluorophore-conjugated anti-mouse antibodies for CD11b, Gr-1, Ly6C (AL-21), and NK1.1 (PK136) (BD BioSciences). Importantly, we showed previously (28) that although the Gr-1 antibody used (clone RB6-8C5) binds both Ly6G and Ly6C epitopes under non-fixed conditions (i.e., for the acute ventilated model), the use of Cytofix/Cytoperm results in loss of Ly6C recognition by the antibody. Therefore, in the context of the resolution model, Gr-1 staining is representative solely of cell Ly6G expression. Cell samples were analyzed by a CyAn flow cytometer with Summit software (Beckman Coulter, High Wycombe, UK), and further data analysis was performed by FlowJo software (Tree Star, Ashland, OR). Absolute leukocyte counts were determined using microsphere beads (Invitrogen, Paisley, UK).

### Statistical Analysis

Statistical analyses were performed using SPSS version 22 (IBM, Portsmouth, UK). The normality of model residuals was assessed by QQ plot and Shapiro–Wilk test. Data that were not normally distributed were transformed (see legends for details), and subsequent parametric distribution confirmed before analyses were carried out. Data that could not be normalized by transformation were analyzed using non-parametric tests. Time-course data in the acute ventilated model were analyzed by repeated measures analysis of variance (ANOVA) followed by pairwise analysis of individual time points, while end-point analyses were carried out by Student’s *t*-test. In the resolving injury model, differences between treatment groups were evaluated on each day, using either Student’s *t*-test or Mann–Whitney *U*-test. A value of *p* < 0.05 was considered significant.

## Results

### Acute Model

**Figure 1:**
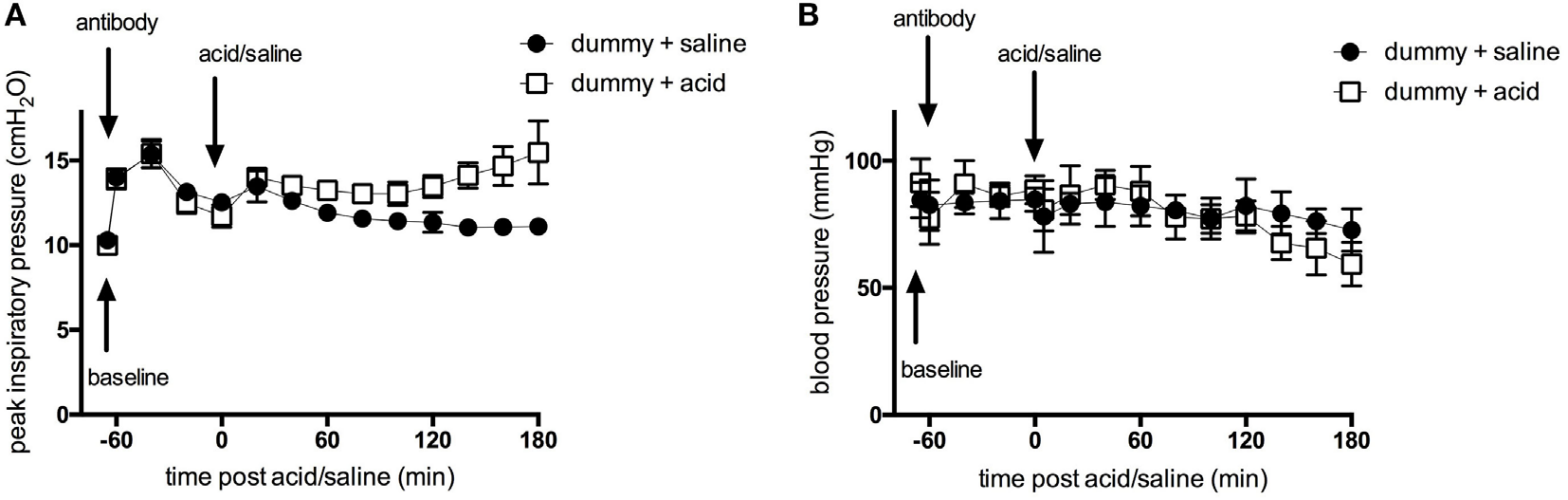
**Peak inspiratory pressure (A) and arterial blood pressure (B) in ventilated animals following intratracheal treatment with dummy dAb, followed by challenge with either intratracheal saline or hydrochloric acid**. *N* = 4 (dummy + saline), or 6 (dummy + acid) at each time point. Data are expressed as mean ± SD.

We first carried out experiments to determine the influence of p55-targeting dAb administration on the acute phase of acid aspiration-induced lung injury. As part of the model development, experiments were performed comparing the physiological consequences of acid instillation versus those of saline instillation. Initial administration of dummy dAb led to a transient increase in peak inspiratory pressure (Figure 1A), which returned toward normal as fluid was distributed and absorbed within the lungs (the same pattern was apparent following administration of p55-targeting dAb; data not shown). Instillation of either saline or acid 60 min later caused similar increases in airway pressure. In animals receiving saline, peak inspiratory pressure decreased and plateaued. In contrast, animals that received acid instillation showed an initial improvement in airway pressure, which then deteriorated over the final 60 min. Intratracheal administrations also caused small, transient decreases in blood pressure, most likely secondary to the sustained inflation maneuvers carried out to distribute instilled fluids (Figure 1B). Blood pressure was otherwise well maintained throughout the experiments until the final 60 min during which it deteriorated somewhat, particularly in acid-treated animals.

**Figure 2:**
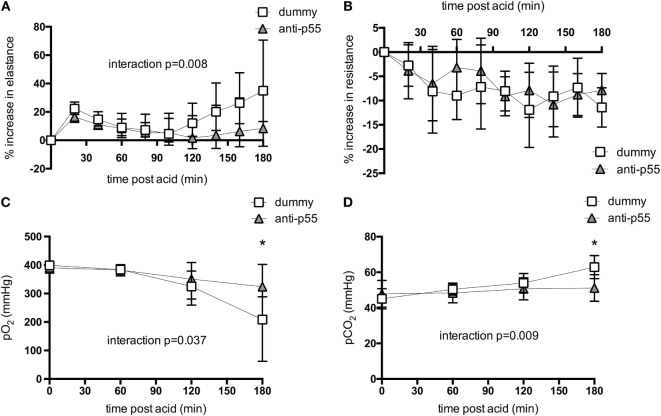
**Respiratory system elastance (A) and resistance (B), and arterial pO_2_ (C) and pCO_2_ (D) following acid instillation in animals treated with dummy or p55-targeting dAb**. Mechanics data are expressed as % increase following acid. Elastance data **(A)** were log-transformed to achieve normal distribution, while pO_2_ **(C)** was normalized by raising to the power of 2. These data are displayed as back-transformed mean with error bars representing 90% confidence intervals. Resistance data and pCO_2_ were normally distributed and thus displayed as mean ± SD. Repeated measures analysis of variance revealed significant interactions between treatment and time for elastance change (*p* < 0.01), pO_2_ (*p* < 0.05), and pCO_2_ (*p* < 0.01). *p < 0.05 between dummy and dAb-treated animals at 180 min after acid. *N* = 5–6 observations from independent experiments at each time point.

The physiological consequences of acid instillation were then compared between animals pretreated with either the dummy non-targeting dAb or the p55-targeting dAb (*N* = 6 independent experiments/group). The initial mechanics response to acid was similar between the two groups (Figure 2A), consisting of a transient spike in elastance followed by a return toward pre-instillation levels. In the dummy dAb group, elastance then increased from ~100 min until the end, whereas in contrast, the p55-targeting dAb-treated mice showed little increase, resulting in a significantly attenuated elastance change (*p* value for interaction <0.01, although pairwise analysis did not detect significant differences at individual time points). Respiratory system resistance showed similar patterns in both groups, consisting of a decrease following acid instillation that remained relatively stable thereafter (Figure 2B). There was also a decrease in arterial pO_2_ and an increase in pCO_2_ during the final hour of ventilation in dummy-treated animals (Figures 2C,D). These impairments in gas exchange were significantly attenuated by treatment with the p55-targeting dAb (*p*-value for interaction <0.05, with pairwise analysis showing significant difference at the 180 min time point).

**Figure 3:**
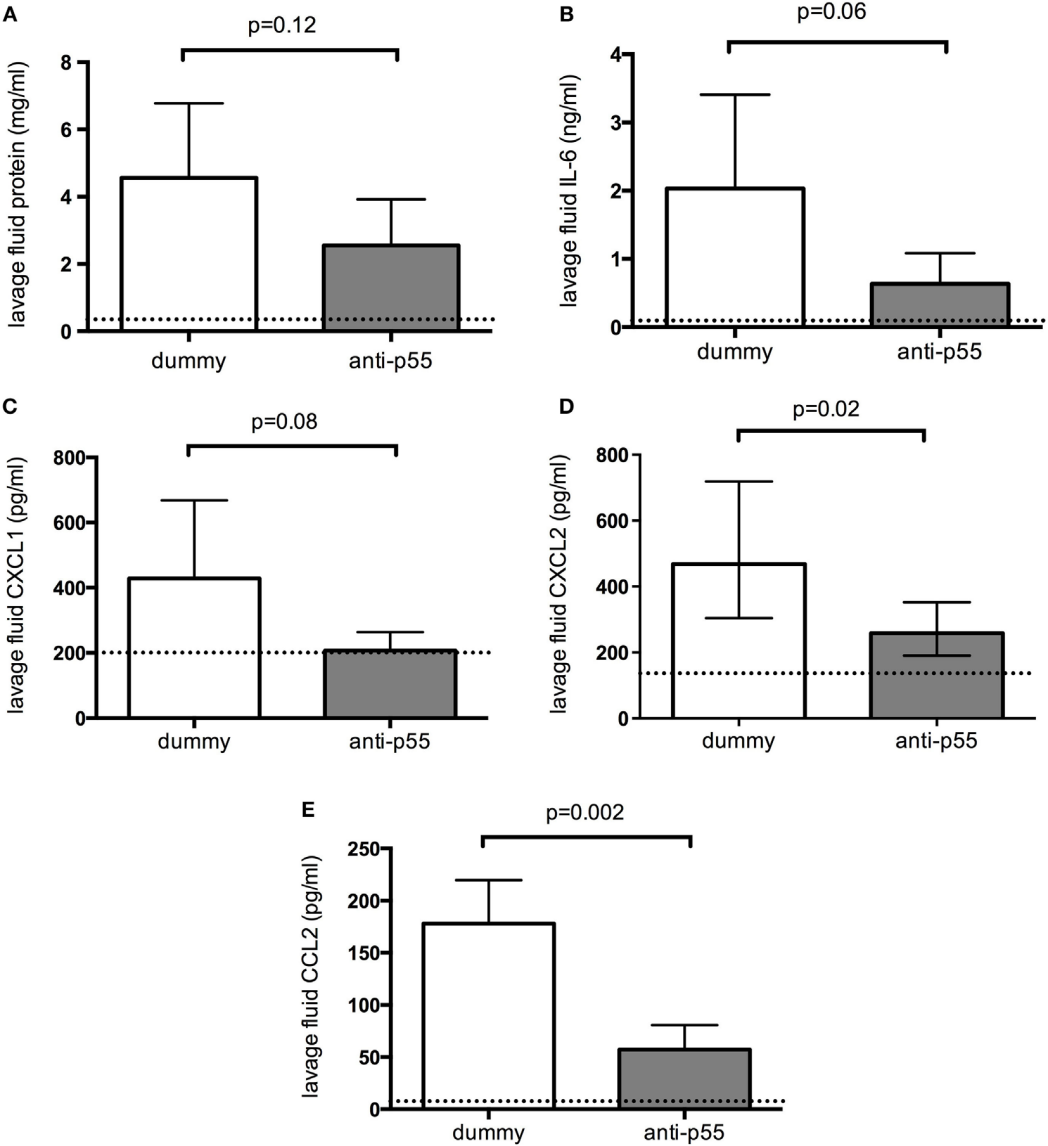
**Lavage fluid levels of total protein (A), interleukin 6 (B), CXCL1 (C), CXCL2 (D), and CCL2 (E) 180 min after acid instillation in animals treated with dummy or p55-targeting domain antibody**. Dotted lines represent data from saline-treated animals for visual comparison. Lavage fluid CXCL2 (D) required log-transformation to normal distribution and is therefore displayed as geometric mean with error bars representing 90% confidence intervals. All other data were normally distributed and thus are displayed as arithmetic mean ± SD. *T*-tests were used to evaluate differences between treatments. *N* = 5 observations from independent experiments for each group (corresponding to the total number of mice assessed for these parameters).

Lung lavage fluid in the dummy-treated group contained a substantial amount of protein, indicating an increase in epithelial/endothelial barrier permeability (Figure 3A, dotted line represents data from saline-treated animals for visual comparison). This was somewhat, though not significantly, reduced by p55-targeting dAb. Evaluation of pro-inflammatory cytokines in lung lavage fluid showed that IL-6 and CXCL1 showed a tendency to be reduced, while CXCL2 and CCL2 were significantly attenuated in p55-targeting dAb-treated mice (Figures 3B–E).

**Figure 4:**
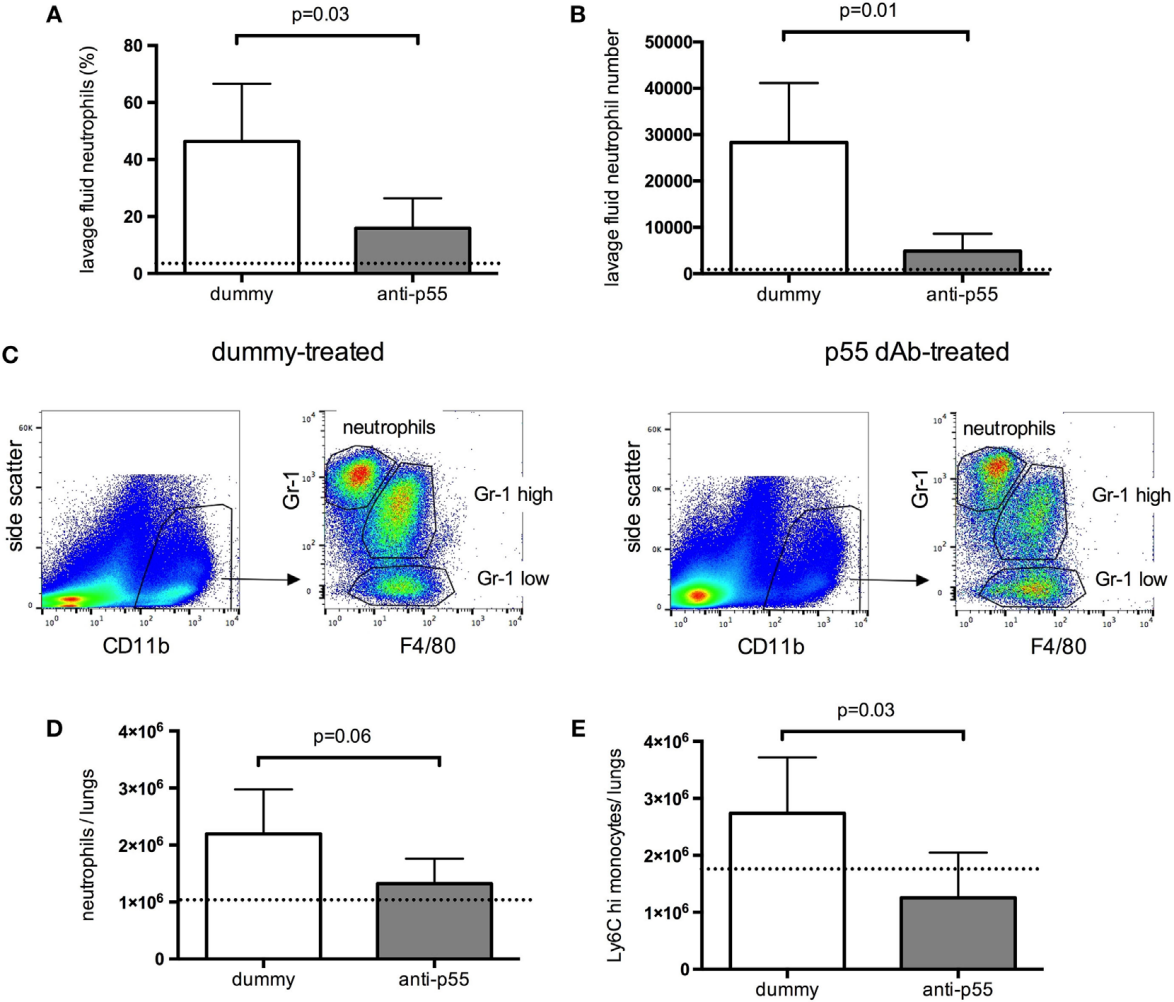
**Recruitment of leukocytes into the alveolar space (A,B), and lung tissue (C–E), 180 min after acid instillation in animals treated with dummy or p55-targeting domain antibody**. For the acute ventilated model, tissue neutrophils were identified as CD11b^high^, Gr-1^high^, F4/80 negative events, while monocytes were identified as CD11b^high^, F4/80 positive events, and differentiated by their expression of Gr-1. Under the non-fixed conditions used for these experiments, the Gr-1 antibody used (clone RB6-8C5) binds both Ly6G and Ly6C epitopes. Panel **(C)** shows side-by-side representative flow cytometric plots for lungs from dummy dAb and p55-targeting dAb-treated animals. Cell numbers were determined by use of fluorescent counting beads and expressed as total cell counts in both lungs **(D,E)**. Data are displayed as mean ± SD, with *t*-tests used to evaluate differences. *N* = 4–5 observations from independent experiments for each group (corresponding to the total number of mice assessed for these parameters).

Finally, lung leukocyte recruitment was determined in each group of mice. Neutrophil infiltration into the alveolar space (evaluated by microscopic cytology of lung lavage fluid) was significantly attenuated by p55-targeting dAb (Figures 4A,B). Total lung tissue neutrophil and monocyte recruitment were evaluated by flow cytometry (Figure 4C). Neutrophil recruitment was somewhat (though not significantly) reduced following p55-targeting dAb, while inflammatory Ly6C^hi^ monocyte numbers were significantly attenuated (Figures 4D,E).

### Resolving Model

While the data from the acute model indicate that intratracheal inhibition of p55 using the dAb attenuates both pulmonary edema and inflammation during the early phase of ALI/VILI, it does not necessarily follow that this would translate to a prolonged benefit. We therefore carried out experiments to investigate the consequences of p55-targeting dAb on the later phase of ALI using the resolving model.

**Figure 5:**
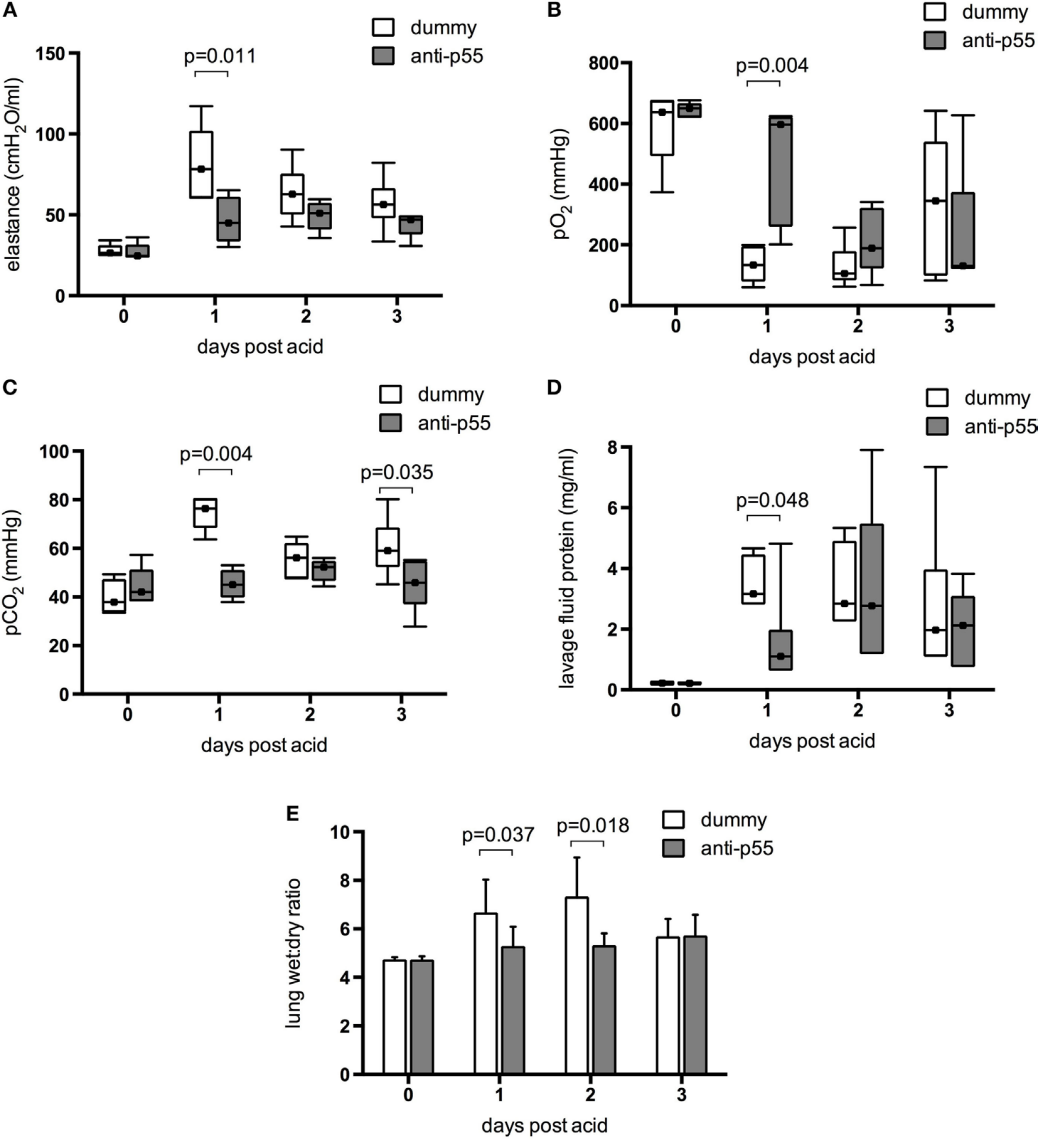
**Physiological indications of injury in the resolution model were determined in terms of elastance (A), arterial pO_2_ (B), and pCO_2_ (C) measured after 30 min ventilation with 100% O_2_**. Data were evaluated for differences due to treatment on each day. Data did not achieve normal distribution even with transformation, and thus are displayed as box-whisker plots and analyzed using Mann–Whitney *U*-test. Error bars extend to maximum and minimum values, while markers within boxes show the median. Physiological parameters of injury were significantly attenuated by p55-targeting dAb treatment at day 1, but this was mostly lost by day 2. *N* = 5–8 for elastance and 5–7 for blood gases at each time point. Permeability/edema was evaluated by lavage fluid protein **(D)** and wet:dry weight ratio **(E)**. Lavage fluid protein is displayed as box-whisker plots and was analyzed using Mann–Whitney *U*-test, while wet:dry weight is displayed as mean ± SD and was analyzed using Student’s *t*-test. *N* = 5–7 for lavage protein and 5–8 for wet:dry ratio at each time point.

Acid instillation caused a clear physiological lung injury in dummy dAb-treated animals, consistent with previous reports using this model (21, 29). Respiratory system elastance (determined after 30 min of ventilation) was increased at 24 h after acid, and subsequently returned toward baseline although did not reach normal values by day 3 (Figure 5A). Similarly, arterial pO_2_ was substantially decreased (Figure 5B) and pCO_2_ increased (Figure 5C) 1 day after acid. pO_2_ remained low at day 2 and then started to recover at day 3, while pCO_2_ showed some signs of return toward normal at day 2. The single pretreatment with p55-targeting dAb led to a significant attenuation in each of these parameters at 24 h. It was clear however that by 48 h most of the protection afforded by the dAb was lost, with animals appearing as injured as dummy dAb-treated mice. Acid instillation also caused increases in pulmonary edema/permeability, assessed by lavage fluid protein (Figure 5D) and lung wet:dry weight ratio (Figure 5E). Both of these markers peaked around days 1–2 before returning toward (but not achieving) baseline levels by day 3. Pretreatment with the p55-targeting dAb reduced lavage fluid protein levels (again, only at day 1) and lung wet:dry ratio (at days 1 and 2).

**Figure 6:**
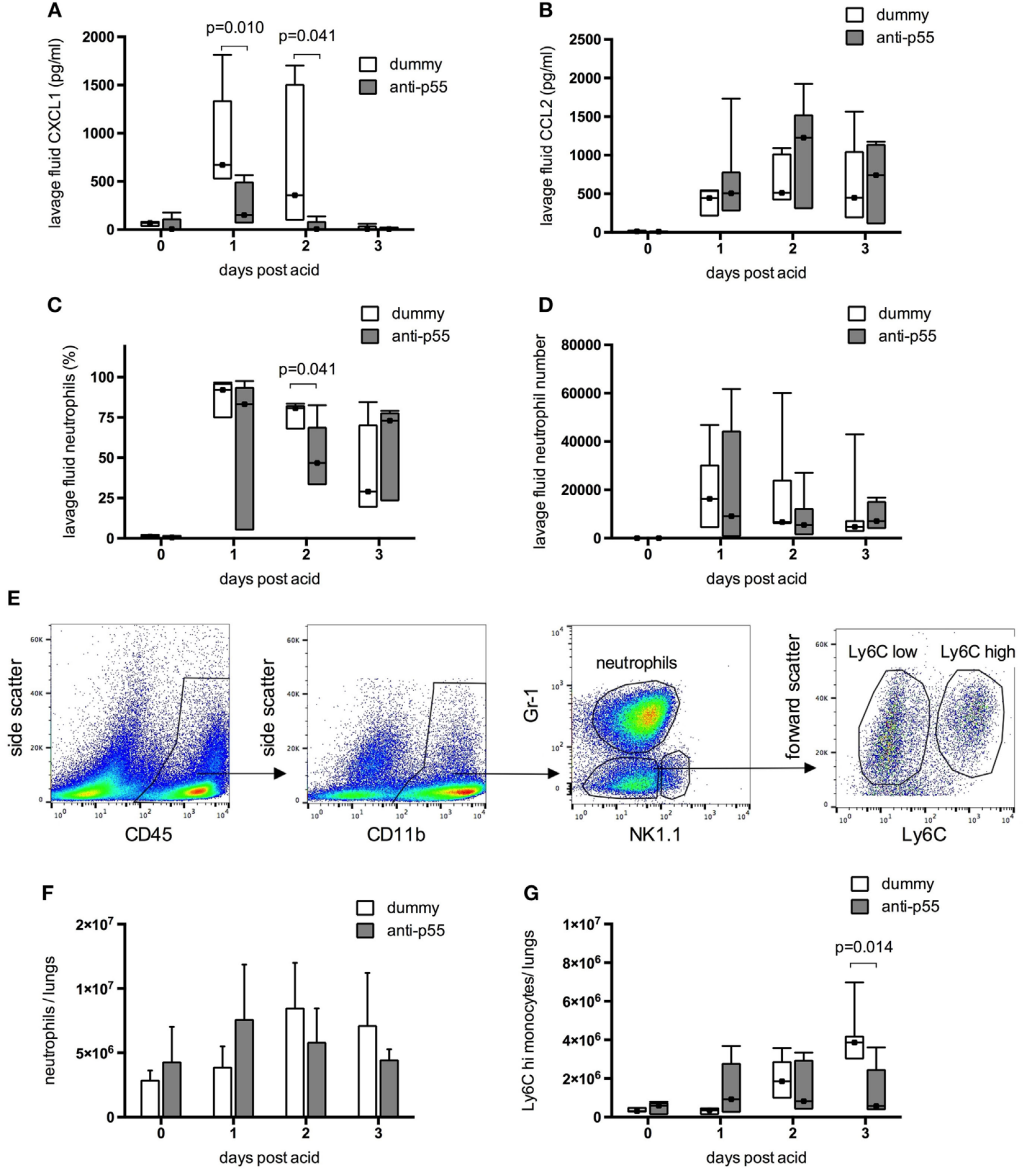
**Inflammation within the resolution model was evaluated in terms of lavage fluid cytokines and leukocyte recruitment**. Data were evaluated for differences due to treatment on each day. Lavage fluid CXCL1 **(A)** and CCL2 **(B)** could not be normalized and are displayed as box-whisker plots with analysis by Mann–Whitney *U*-test (*N* = 4–7 at each time point). CXCL1 levels were significantly attenuated following pretreatment with p55-targeting dAb, while CCL2 was unaffected. Neutrophil infiltration into the alveolar space **(C,D)** was highly variable. Neutrophil percentage was reduced at day 2 by dAb treatment, although numbers recruited were not significantly attenuated. For the resolution model neutrophils, monocytes, and NK cells within lung tissue were identified as CD45 positive, CD11b^high^ events using flow cytometry, and differentiated by their expression of Gr-1 and NK1.1 [representative plots from dummy-treated mouse at 24 h after acid shown in panel **(E)**]. NK cells were identified as NK1.1 positive events. We showed previously (28) that although the Gr-1 antibody used (clone RB6-8C5) binds both Ly6G and Ly6C epitopes under non-fixed conditions, the use of Cytofix/Cytoperm results in loss of Ly6C recognition by the antibody. Therefore, in the current context, Gr-1 staining is representative of cell Ly6G expression. Within the NK1.1 negative events, neutrophils were thus identified as Gr-1 (Ly6G) high events, while Gr-1 (Ly6G) low events were designated as monocytes. These were further subcategorized based on expression of Ly6C. Total lung tissue recruitment of neutrophils **(F)** and Ly6C^hi^ monocytes **(G)** were not different following dummy or p55-targeting dAb treatment, apart from a reduction in Ly6C^hi^ monocytes at day 3. Neutrophil numbers in lung tissue **(F)** are displayed as mean ± SD, while Ly6C^hi^ monocyte numbers **(G)** and neutrophils in lavage fluid **(C,D)** could not be normalized and are displayed as box-whisker plots (evaluated using Mann–Whitney *U*-test). *N* = 5–7 for each time point.

Finally, the effect of p55-targeting dAb on inflammation within the lungs was evaluated after acid instillation. Lavage fluid CXCL1 (Figure 6A) was increased to a variable degree in dummy dAb-treated animals, but still significantly attenuated at days 1 and 2 following p55-targeting dAb. In contrast, levels of CCL2 (Figure 6B) were similar between the treatment groups on all days, and unlike CXCL1, did not return to baseline by day 3. Neutrophil infiltration into the alveolar space (by differential cytology) was highly variable and showed little difference between treatment groups (Figures 6C,D), apart from a small reduction in neutrophil percentage at day 2 following p55-targeting dAb. Lung tissue recruitment of neutrophils and Ly6C^hi^ monocytes, evaluated by flow cytometry (Figure 6E), showed large increases between 24 and 48 h after acid in dummy dAb-treated animals (Figures 6F,G), consistent with previously published kinetics in this model of injury (30). However, there was no significant difference in cell recruitment with p55-targeting dAb, apart from a reduction in Ly6C^hi^ monocytes at day 3.

## Discussion

Despite much research, and major advances in our understanding of the pathophysiology, effective pharmacological therapies for ARDS patients remain elusive. It is generally accepted that inflammation plays an important role in ARDS, and numerous drug targets have been identified within preclinical settings, but none of these have translated into clinical treatment. The reasons for this have been widely discussed, including the recent debate regarding the similarity of inflammatory responses between rodents and humans (31, 32), and the possible different responses of ARDS subphenotypes (33). Perhaps more to the point is that animal models of ARDS generally replicate a limited number of the features seen in patients (24, 25). Many models are either too severe/invasive to explore longer consequences (i.e., animals cannot be studied beyond the acute phase) or too mild to replicate the complex pathology. Thus, these models often have very limited predictive power, particularly when they are considered in isolation, in terms of the consequences of inhibiting pathways. For this reason, in the current study, we explored the impact of a domain antibody (dAb™) targeting the p55 TNFR, which we previously showed to be efficacious in attenuating VILI in mice (19), in more clinically relevant acute and resolving models of acid aspiration-induced lung injury.

In the acute ventilated model, the data demonstrate a very clear attenuation of injury in the animals receiving p55-targeting dAb. Specifically, respiratory mechanics and blood gases were significantly preserved. While pairwise analysis of individual time points indicated that only pO_2_ and pCO_2_ showed significant differences, and then only at the final time point, repeated measures ANOVA demonstrated clear interaction effects for elastance, pO_2_ and pCO_2_. Given that the repeated measures ANOVA both maximizes the data being utilized within any given analysis and decreases the chance of type II error by reducing within group variability, these data show that the development of “physiological injury” over time was attenuated by the administration of p55-targeting dAb. We did not evaluate lung histology in these animals, although respiratory system mechanics and blood gasses are clinically important parameters crucial for the diagnosis of ARDS, which are not always evaluated within animal models. We have previously evaluated histological changes within the acute and resolving acid-induced injury models (18, 21) and showed that it correlates well with these other markers. In addition to the changes observed, lung permeability showed a tendency toward protection. Overall, these data are consistent with our previous investigations into acid-induced lung injury in genetically modified animals (18), confirming the importance of the p55 TNFR pathway in this model of pulmonary injury and edema formation. Interestingly, in that previous study, we found that “classic” inflammatory mediators (IL-6, CXCL1, CCL2) and leukocyte recruitment were unaffected by the absence of p55 signaling (18), while here we found quite clearly that the acute intratracheal inhibition of p55 using the dAb led to significant attenuation of alveolar cytokine/chemokine levels, and reduced recruitment of neutrophils and inflammatory Ly6C^hi^ monocytes. The reasons for this apparent difference may relate to consequences of compartmentalized inhibition of p55 signaling versus whole body absence of p55, or the effects of chronic compensation of signaling pathways in genetically modified animals. A similar phenomenon is apparent when comparing genetic modification versus acute inhibition of p55 in models of VILI (17, 19) so although the underlying reasons are unclear, they are not model specific. An alternative explanation could be that the dAb has some additional “off-target” effects. We believe this is unlikely, as binding data demonstrated that dAb has high affinity binding to the p55 TNFR and no specific binding to the closely related p75 receptor. This could be clarified in future experiments by exploring the consequences of p55 dAb administration into p55 knockout mice.

A major aspect of the current study was to explore the influence of p55 inhibition beyond the very acute (3–4 h) phase; it may be dangerous to assume that any early consequences of p55 inhibition will continue to be beneficial into the later stages of disease progression, without understanding the knock-on effects of interfering with this pathway. For this reason, we utilized a more chronic model of acid aspiration-induced injury developed within our research group (21). For the purposes of this study, we investigated the first 72 h after injury, a time frame that from our previous work encompasses the peak of injury and beginnings of a return toward homeostasis. In order to avoid frequent airway manipulation, we chose to use the intranasal route for delivery of domain antibody, followed by intratracheal acid instillation. Studies have estimated that the intranasal administration technique achieves approximately 50% delivery into the airspaces (34). This, combined with the fact that we were looking for consequences of domain antibody administration over a much longer time period, led us to increase the antibody dose from 25 µg in the acute model to 100 µg. Within these experiments, we found that markers of respiratory system mechanics, blood gases, and edema/permeability were significantly improved by the single pretreatment with p55-targeting dAb. However, for a number of these markers, most notably arterial pO_2_ and lavage fluid protein, the injury induced by acid was delayed rather than prevented. Thus, in general, most of the protective effects of a single dose of p55-targeting dAb treatment in terms of physiology were lost after 24 h.

p55-targeting dAb treatment also influenced inflammatory markers within the chronic model, although these were less pronounced than the physiological findings. Levels of CXCL1 and lavage fluid neutrophils (percentage of alveolar cells) were attenuated at days 1–2, but CCL2 levels were not. Interestingly, there was no clear difference in numbers of lung tissue leukocytes on day 1 (or 2) after acid, at which point physiological injury was clearly attenuated following p55-targeting dAb. Ly6C^hi^ monocyte recruitment was attenuated at day 3, and while these cells have been reported as being injurious in the acute phase of injury (27, 35, 36), the physiological relevance of the current finding is unclear. It is possible that this later phase of monocyte recruitment represents transmigration of a reparative subset (37), although this remains speculation.

Tumor necrosis factor-α, as a highly pleiotropic cytokine, plays a multitude of roles during the pathogenesis of ARDS, and these roles may themselves change during the progression of the syndrome from acute exudative to chronic resolution phases. The data from this study and our previous investigations (17–19) indicate that while TNF p55 signaling is involved in both physiological injury/pulmonary edema formation and lung leukocyte recruitment, there is a clear lack of correlation between these two processes. Other pathophysiological mechanisms mediated by TNF may be more important in determining alveolar epithelial barrier function and fluid balance during ARDS than its role in recruiting leukocytes, which may involve more redundant pathways. Specifically, TNF has a complicated involvement in clearance of pulmonary edema fluid (38). We have previously shown that inhibition of p55 signaling was able to prevent caspase-8 activation within epithelial cells and thus allow maintenance of barrier function and alveolar fluid clearance (18). Inhibition of TNF may also aid fluid clearance by reduction of downstream CXCL1 expression (seen in the current study), the human homolog of which (CXCL8) depresses fluid transport (39). However, TNF has also been shown in opposition to this, to promote clearance of water from the lung via direct activation of epithelial sodium channels (40), prevention of which could be highly damaging. We did not evaluate alveolar fluid clearance in this study, but there was no clear evidence that our treatment regime had adverse effects on physiological parameters, possibly because the fluid clearance promoting activity of TNF seemingly occurs mainly through a receptor-independent pathway (41). Within our acute experiments, the earliest detectable consequence of p55 inhibition occurred around 120 min after acid injury, at which point elastance started to diverge from dummy dAb-treated animals. Future study of such early time points may therefore yield important information regarding the links between TNF, leukocytes, and physiological injury.

In the current study, we chose to use a prophylactic strategy of dosing animals before induction of injury to identify the true potential of p55 inhibition using the dAb. Once their lungs are significantly injured by acid to the level comparable to clinical ARDS, mice do not tolerate anesthesia and intra-airway drug delivery (either intratracheal or intranasal) very well. For this reason, it was not possible to give repeated dosings of antibody, which may have enhanced or prolonged the protective effects observed. The domain antibody used within this study has been formulated specifically for airway administration and has a very short half-life in the circulation, so unfortunately systemic dosing to explore later consequences was also not possible here. Future work may include testing of a recently characterized p55-targeting dAb (DMS5540, GSK), which has been modified to provide an extended circulating half-life (42), although it remains unclear whether this would be able to penetrate into the alveolar space (or indeed whether this would be necessary). While it could be argued that prophylactic dosing in preclinical models of disease may not represent the most clinically relevant scenario, it is perhaps not so unreasonable in the case of ARDS. Recent studies report that up to 75% of patients suffering from ARDS acquired or developed it after entering hospital or during their ICU stay (43, 44). Identification of treatments that work safely when delivered prophylactically to “at risk” patients, or very early during the onset of disease, may therefore be a useful strategy to prevent progression to ARDS, although the same interventions should not be expected to be therapeutically efficacious in patients with established disease.

In conclusion, our data show clearly that targeting of the p55 TNFR with use of an intrapulmonary domain antibody attenuates many physiological indications of acid aspiration-induced lung injury, including respiratory mechanics, blood gases, and markers of edema/permeability. These data show the persistent importance of p55 TNF signaling in both the very acute and later phases of lung injury, and thus strongly support a role for p55 inhibition in patients with or at risk of lung injury, an approach that is currently being developed clinically. Although in the current study the beneficial effects were mainly lost after 24 h of injury, the protection up to this point was achieved with just a single intranasal dosing. Subsequent experiments will be necessary to determine the potential for modified dosing regimes to prolong the attenuation in injury observed.

## Ethics Statement

All animal protocols were approved by the Ethical Review Board of Imperial College London and carried out under the authority of the UK Home Office in accordance with the Animals (Scientific Procedures) Act 1986, UK, and EU Directive 2010/63/EU.

## Author Contributions

MW, MT, PM, and AB were involved in the initial design of the study. MW, KW, SB, CO, BP, KO, and JC were involved in the acquisition and analysis of data. MW, KW, and MT prepared the manuscript. MW, KW, SB, CO, BP, KO, JC, PM, AB, and MT were involved in revising the manuscript and approving it for publication.

## Conflict of Interest Statement

MW and MT received research funding from GlaxoSmithKline to carry out this work. JC, PM, and AB are employed by, and hold stock in, GSK. GSK has a financial interest in the use of domain antibodies, including those targeting p55 TNF receptor, in the treatment of pulmonary and other diseases. GSK was involved in the initial design of the study and contributed to the study report, and produced the domain antibodies and binding kinetic data but had no other involvement in collection, analysis, and interpretation of data, or in the decision to submit the report for publication. Other authors declare that they have no potential conflicts of interest.
